# A NAC transcription factor and its interaction protein hinder abscisic acid biosynthesis by synergistically repressing *NCED5* in *Citrus reticulata*

**DOI:** 10.1093/jxb/eraa118

**Published:** 2020-05-31

**Authors:** Feng Zhu, Tao Luo, Chaoyang Liu, Yang Wang, Li Zheng, Xue Xiao, Mingfei Zhang, Hongbin Yang, Wei Yang, Rangwei Xu, Yunliu Zeng, Junli Ye, Juan Xu, Jianguo Xu, Robert M Larkin, Pengwei Wang, Weiwei Wen, Xiuxin Deng, Alisdair R Fernie, Yunjiang Cheng

**Affiliations:** 1 National R&D Center for Citrus Preservation, Key Laboratory of Horticultural Plant Biology, Ministry of Education, Huazhong Agricultural University, Wuhan, P.R. China; 2 Zhejiang Citrus Research Institute, Taizhou, Zhejiang, China; 3 Max Planck Institute of Molecular Plant Physiology, Potsdam-Golm, Germany; 4 Fondazione Edmund Mach, Italy

**Keywords:** ABA, *Citrus reticulata*, fruit ripening, MYB transcription factor, NAC transcription factor, postharvest, synergistic transcriptional regulation

## Abstract

Although abscisic acid (ABA) is a vital regulator of fruit ripening and several transcription factors have been reported to regulate ABA biosynthesis, reports of the effect of ABA on citrus ripening and the regulation of its biosynthesis by a multiple-transcription-factor complex are scarce. In the present study, a systematic metabolic, cytological, and transcriptome analysis of an ABA-deficient mutant (MT) of *Citrus reticulata* cv. *Suavissima* confirmed the positive effect of ABA on the citrus ripening process. The analysis of transcriptome profiles indicated that CrNAC036 played an important role in the ABA deficiency of the mutant, most likely due to an effect on the expression of *9-cis-epoxycarotenoid dioxygenase 5* (*CrNCED5*). Electrophoretic mobility shift assays and dual luciferase assays demonstrated that CrNAC036 can directly bind and negatively regulate *CrNCED5* expression. Furthermore, yeast two-hybrid, bimolecular fluorescence complementation, and dual luciferase assays demonstrated that CrNAC036 interacted with CrMYB68, also down-regulating the expression of *CrNCED5*. Taken together, our results suggest that CrNAC036 and CrMYB68 synergistically inhibit ABA biosynthesis in citrus fruit by regulating the expression of *CrNCED5*.

## Teaser

A novel NAC transcription factor, CrNAC036, can interact with CrMYB68 to synergistically regulate the ripening process of citrus fruit by down-regulating the expression of *9-cis-epoxycarotenoid dioxygenase 5*.

## Introduction

Fruit ripening is a complex process, and based on the patterns of respiration and ethylene biosynthesis during fruit ripening, fleshy fruits can be divided into two categories, namely, climacteric and non-climacteric fruits (Giovannoni, 2004). Abscisic acid (ABA) is a vital hormone that affects the ripening process of both categories. Knocking-down *9-cis-epoxycarotenoid dioxygenases* (*NCEDs*) led to a significant down-regulation of the expression of genes associated with cell wall catabolism (such as *pectate lyase* and *expansin*) in tomato. Moreover, the application of exogenous ABA or treatment with an inhibitor of its biosynthesis can significantly promote or hinder the color change, softening, and flavor component accumulation of tomato, peach, strawberry, and grape fruits (Zhang et al., 2009*a*,*b*; Jia *et al.*, 2011; Sun *et al.*, 2012; Leng *et al.*, 2014). Beside the effect on fruit ripening, exogenous ABA treatment can also induce the expression of genes associated with chlorophyll degradation (such as *stay-green* and *pheophytinase*) and promote the senescence of Arabidopsis (Yang *et al.*, 2014). Owing to ABA’s vital role in fruit ripening, the pathway of its biosynthesis has been well characterized: violaxanthin derived from carotenoid metabolism is transformed by ABA4, NCEDs, and several other enzymes to yield ABA (Finkelstein, 2013). As the rate-limiting enzymes of ABA biosynthesis, NCEDs have attracted considerable of attention and the transcriptional regulation of *NCEDs* is one of the hot topics in ABA research. In tomato, banana, peach, and citrus fruit, WRKYs, ERF3, and bHLH1 transcription factors can directly bind to their promoters and regulate the expression of *NCEDs* (Endo *et al.*, 2016; Luo *et al.*, 2017; Wang *et al.*, 2019b). In the well-known model of anthocyanin biosynthesis, the synergistic transcriptional regulation of the MBW complex (MYB–bHLH–WD40) can induce higher expression of anthocyanin-associated genes than the effect of the single transcription factors separately, which demonstrated that synergistic regulation of transcription factor complexes is more efficient in the fruit ripening process (Schaart *et al.*, 2013; Wang *et al.*, 2019a). However, reports concerning the synergistic transcriptional regulation of multiple transcription factors on fruit *NCEDs* remain limited.

The regulatory mechanism of fruit ripening originated from those of carpel senescence. The carpel senescence-related NAC (*N*AM/*A*TAF/*C*UC) transcription factors play vital roles in different fruit ripening processes (Lü *et al.*, 2018). SlNOR-like1 and SlNAC4 can act as positive regulators of tomato color formation and several NACs play important roles in apple and oil palm fruit development (Zhu *et al.*, 2014; Tranbarger *et al.*, 2017; Gao *et al.*, 2018; Zhang *et al.*, 2018). Moreover, NAC proteins can also directly control the monoterpene synthesis of kiwifruit and the lignification of postharvest loquat fruit (Nieuwenhuizen *et al.*, 2015; Xu *et al.*, 2015). In order to enhance the regulatory effect on the downstream genes, NAC transcription factors often form homodimers or heterodimers with other proteins. In peach, the NAC transcription factor BL can interact with NAC1 to amplify its regulatory effect on anthocyanin biosynthesis; and in banana fruit, NAC5 can interact with WRKY1/2 to enhance the induction of resistance genes (Zhou *et al.*, 2015; Shan *et al.*, 2016). Besides the positive effect of NAC regulators on ripening and senescence, overexpressing the NAC transcription factors *JUNGBRUNNEN1* (*JUB1*, *ANAC042*) and *VNI2* (*ANAC083*), and *TaNAC-S* significantly delayed the senescence of Arabidopsis and wheat seedlings (*Triticum aestivum* L.), respectively (Yang *et al.*, 2011; Wu *et al.*, 2012; Zhao *et al.*, 2015). This fact notwithstanding, little is known concerning the negative regulatory effects of NAC on fruit ripening.

Citrus is one of the most important fleshy fruit crops in the world. However, research on this important crop is restricted by its long juvenile phase, high heterozygosity and the difficulty in obtaining transgenic plants. Fortunately, natural mutants can help researchers to overcome these limitations. In the past decades, experiments with ABA-deficient natural mutants and ABA treatments have indicated that ABA is a vital regulator of color change and the metabolism of both sugars and organic acids (Rodrigo *et al.*, 2003; Wu *et al.*, 2014; Zhang *et al.*, 2014; Rehman *et al.*, 2018). Recently, we found a novel stay-green natural mutant (MT) in *Citrus reticulata* cv. *Suavissima*, and used it to study the transcriptional regulation of CrMYB68 on carotenoid metabolism via effects on *CrBCH2* and Cr*NCED5* (Zhu *et al.*, 2017). However, research concerning comprehensive effects of ABA on the ripening process (such as chlorophyll degradation, chloroplast disassembly, and ripening-related gene expression) and synergistic transcriptional regulation of ABA biosynthesis in citrus is rare. In the present study, we comprehensively analysed the effect of ABA on citrus ripening and further demonstrated that a novel NAC transcription factor, CrNAC036, directly regulated the expression of *CrNCED5* and could additionally interact with CrMYB68 to synergistically down-regulate the expression of *CrNCED5*, providing mechanistic insight into the low ABA content in MT.

## Materials and methods

### Plant materials

Fruit from *Citrus reticulata* cv. *Suavissima* (wild type, WT) and its spontaneous stay-green mutant (mutant type, MT) were harvested from trees in the same commercial orchard in Wenzhou (Zhejiang Province, P.R. China) at 120 (enlarged stage), 170 (mature green stage), 210 (commercially ripe stage), and 245 (fully ripe stage) days after flowering (DAF) in 2011 and 2012. Given that we only observed slight phenotypic differences between WT and MT at 120 DAF and intended to obtain more information about the fruit at the breaker and postharvest stages, fruit samples were collected at 170, 185 (the breaker stage), and 210 DAF and 30 d after storage (DAS) in 2013.

### Treatments and sampling

Both MT and WT fruits were harvested at 170 DAF and randomly divided into two groups. MT and WT group I fruits were dipped into 100 μM ABA solution (132 mg ABA was first dissolved in 1 ml ethanol and then added to 5 liters of distilled water containing 0.1% Tween 80) for 2 min. MT and WT group II fruits were dipped into 5 liters of distilled water (containing 1 ml ethanol and 0.1% Tween 80) for 2 min. After drying at room temperature for 30 min, the fruits were transferred to a sealed chamber (temperature 20–25 °C and relative humidity 75–85%) and sampled at 0 and 6 h after treatment. The concentration and the method of ABA treatment were according to former reports (Zhang *et al.*, 2009*b*; Oh *et al.*, 2018). All sampled tissues were immediately frozen in liquid nitrogen, powdered, and stored at −80 °C until analysis.

### Fruit storage and sampling

The fruits harvested at 210 DAF in 2013 were used for a storage experiment. They were packed in plastic bags and kept at the optimum cold storage temperature (15–18 °C) and relative humidity (75–85%). At 30 DAS, the flavedo was sampled, frozen in liquid nitrogen, and immediately stored at −80 °C.

### Extraction and UPLC analysis of chlorophylls and chlorophyll derivatives

Chlorophylls and chlorophyll derivatives were extracted according to a method described by Minguez-Mosquera and Garrido-Fernandez (1989). The separation and quantification of chlorophylls and derivatives were carried out by a UPLC (Waters, H-Class) system using a C18 column (BEH C18, 50×2.1 mm, i.d. 1.8 μm). Separation was performed using an elution gradient (0.4 ml min^−1^) with the mobile phases (A: water: ion pair reagent: methanol (1:1:8, v/v/v), B: methanol: acetone (1:1, v/v)) as described by Mínguez-Mosquera *et al.* (1991). The elution gradient program was optimized as follows (time, A): 0 min, 75%; 3 min, 35%; 4 min, 35%; 5 min, 25%; 6 min, 16%; 8 min, 0%; 9 min, 75%; 0 min, 75%. The on-line UV-visible spectra were recorded from 350 to 750 nm with a photodiode array detector (eλ PAD). Detection was at 654 nm for chlorophyll *b* and its derivatives and 664 nm for chlorophyll *a* and its derivatives. Data were collected and processed with Epower 3 software. Chlorophylls and their derivatives were identified by comparing their retention time and spectral characteristics with those of authentic standards. At least three independent extractions and detection were performed for each sample.

### Analysis of soluble sugars and organic acids by gas chromatography

Contents of soluble sugars and organic acids were determined using gas chromatography. The samples were frozen with liquid nitrogen and powdered. A total of 1 g of frozen powder was analysed by gas chromatography as described previously (Wu *et al.*, 2014) with minor modification. The powder was suspended in 8 ml pre-cooled 80% methanol and incubated in a 70 °C water bath for 30 min. After a 1.5 h ultrasonic extraction and centrifugation at 4000 *g* for 10 min, the supernatant was collected and 0.2 ml internal standard (2.5% w/v phenyl-β-D-glucopyranoside, 2.5% w/v methyl-α-D-glucopyranoside) was added. The solution was made up to 50 ml with 80% methanol, and a 1 ml aliquot of this final supernatant was vacuum-dried. The dried sample was re-dissolved in 800 μl 2% w/v hydroxylamine hydrochloride in pyridine at 70 °C for 1 h and then 400 μl hexamethyldisilazane and 200 μl trimethylchlorosilane were added for incubation at 70 °C for 2 h; 0.5 μl of the supernatant was analysed with an Agilent 6890 N device (Santa Clara, CA, USA) equipped with a flame ionization detector. Sugars and organic acids were identified through a comparison of retention times using standard compounds from Sigma-Aldrich (St Louis, MO, USA).

### Transmission electron microscopy

The flavedo from the fruit harvested without damage at 170, 185 and 210 DAF and 30 DAS was analysed using transmission electron microscopy as described previously (Cao *et al.*, 2012) with minor modification. The flavedos of MT and WT fruits were fixed with 2.5% glutaraldehyde and 0.1 M phosphate buffer with 2% OsO4. The fixed samples were dehydrated in epoxy resin and embedded in SPI-812. Ultrathin sections obtained with a Leica UC6 ultramicrotome were stained with uranyl acetate and subsequently with lead citrate. The images were captured by a HITACHI H-7650 transmission electron microscope at 80 kV and a Gatan 832 CCD camera.

### Western blot analysis

Total proteins were extracted as described previously (Cao *et al.*, 2012), and quantified using a RC DC protein assay kit (Bio-Rad, Hercules, CA, USA). Then, 30 μg of total flavedo protein was resolved by SDS-PAGE (12.5%) and transferred to polyvinylidene fluoride membranes (Millipore, USA). The subsequent western blot analysis was conducted as described previously (Cao *et al.*, 2012), using the following primary antibodies (1:3000, v/v): rabbit anti-Lhca1, anti-Lhca2, anti-Lhca3, anti-Lhca4 and anti-Lhcb1 (Agrisera, Sweden); and the following secondary antibodies (1:15 000, v/v): peroxidase-conjugated immunopure goat anti-rabbit or goat anti-mouse IgG [H+L] (Pierce, USA). Signals were detected using a Clarity Western ECL Substrate (Bio-Rad) according to the manufacturer’s instructions. The chemiluminescence signal was imaged using a ChemiDoc XRS (Bio-Rad) and quantified using Quantity One software (Bio-Rad). The calculated intensity volumes were fitted with a variable slope dose–response relationship using ImageJ.

### RNA isolation and comparative quantitative real time PCR analysis

Total RNA was extracted as described previously (Cao *et al.*, 2012). The integrity of the RNA preparations was evaluated by electrophoresis and then their concentrations, *A*_260_/*A*_280_ ratios and *A*_230_/*A*_260_ ratios were determined using a Nanodrop spectrophotometer (Agilent 2100, USA). Two biological replicates of RNA from the flavedo of MT and WT (170DAF) treated with ABA were hybridized to GeneChip^®^ Citrus Genome Arrays (Affymetrix^®^; Santa Clara, CA, USA). The analyses of the gene annotation and differentially expressed genes (DEGs) were performed as described previously and DEGs were detected with restriction of *P*>0.01 and fold-change greater than 2 (see Supplementary Table S1 at *JXB* online) (Ma *et al.*, 2014b). The transcriptome datasets generated using the GeneChip^®^ Citrus Genome Array platform can be found in the Gene Expression Omnibus (GEO) with the accession number GSE113669. Genes and primers used for the quantitative reverse transcription-PCR analysis are listed in Supplementary Table S2.

### Isolation and analysis of *CrNAC036* sequence

The coding sequences of *CrNAC036* were amplified from cDNA using gene-specific primers (see Supplementary Table S2). The Clustal W program and GeneDoc software were used to align and edit the different amino acid sequences. Using the neighbor-joining algorithm, a phylogenetic tree was constructed with the amino acid sequence of CrNAC036 and those of 105 NAC transcription factors from Arabidopsis using MEGA 5.0 (Tamura *et al.*, 2011). Bootstrap analysis was performed using 1000 replicates in MEGA 5.0 to evaluate the reliability of the different phylogenetic group assignments. The respective names and TAIR ID numbers of the 105 NAC sequences are presented in Supplementary Table S3.

### Subcellular localization of CrNAC036

The subcellular localization of the CrNAC036 protein was determined as described previously (Zhu *et al.*, 2017). Protoplasts were co-transformed with *35S:CrNAC036*-pM999-GFP and the nuclear marker vector *35S:OsGhd7*-CFP. Fluorescence from green fluorescent protein (GFP) and cyan fluorescent protein (CFP) was observed using a confocal laser-scanning microscope (TCS SP2, Leica, Germany). The excitation and emission filters used to detect fluorescence from GFP were 488 nm and 500–530 nm, respectively. The excitation and emission filters used to detect signals from CFP were 430 nm and 470–510 nm, respectively. Chlorophyll autofluorescence was monitored using the excitation wavelength of either 488 or 514 nm and the emission wavelengths from 650 to 750 nm.

### Protein preparation, identification, and electrophoretic mobility shift assay

pET15 (Novagen) was used to produce a recombinant CrNAC036 protein with a 6×His tag fused to the N-terminus. *Escherichia coli* strain BL21 (DE3) was used to express the recombinant CrNAC036 protein. We purified and characterized the recombinant protein as previously described (Zhu *et al.*, 2017) with minor modification. The recombinant protein was analysed by matrix-assisted laser desorption/ionization time-of-flight tandem mass spectrometry (5800 MALDI-TOF/TOF, AB SCIEX) with a mass spectrometer to acquire MALDI and MS/MS spectra after tryptic digestion. The MS spectra were recorded in reflector mode with a mass range of 800–4000. In MS/MS positive ion mode, for one main MS spectrum, 50 subspectra with 50 shots per subspectrum were accumulated using a random search pattern. Collision energy was 2 kV and the collision gas was air. The database search was performed using the MASCOT search engine 2.2 (Matrix Science, Ltd) embedded into GPS-Explorer Software 3.6 (Applied Biosystems) against non-redundant protein databases of *Citrus clementina* (https://phytozome.jgi.doe.gov/pz/portal.html#!info?alias=Org_Cclementina). Additionally, MS/MS fragment tolerance was set to 0.4 Da. A protein confidence index ≥95% was used for further manual validation.

An electrophoretic mobility shift assay (EMSA) was performed as previously described (Zong *et al.*, 2016; Zhu *et al.*, 2017). Briefly, the His-tagged CrNAC036 protein and 5′-FAM-labeled oligonucleotide probes (synthesized by the Shanghai Sangon Company) were incubated in a binding solution (0.1% Nonidet P-40, 1 mM benzamidine, 0.5 mM phenylmethylsulfonyl fluoride, 0.5 mM DTT, 50 μg ml^−1^ BSA and 100 ng μl^−1^ poly (dI-dC)) at 4 °C for 45 min. For the competition assays, after the protein was incubated with non-labeled probe at 4 °C for 45 min, 1 µl of the 5′-FAM-labeled probe (10 µmol l^−1^) was added to the mixture and incubated at 4 °C for 45 min. The binding reactions were resolved using electrophoresis with 6% polyacrylamide gels at 4 °C in 0.5×TBE (Tris-Borate-EDTA) in the dark for 1 h and imaged with an Amersham TM Imager 600 (GE Healthcare).

### Dual luciferase and bimolecular fluorescence complementation assays

We used rice protoplasts for the dual luciferase transcriptional activity assay as described previously (Zong *et al.*, 2016) because of the high stability, transformation efficiency, and short growth cycle of rice. The Dual-Luciferase^®^ Reporter Assay System (Promega) was used to measure the luciferase activity according to the manufacturer’s instructions. The relative luciferase activity was calculated as the ratio of firefly luciferase (fLUC)/*Renilla* luciferase (rLUC).

To prevent chlorophyll fluorescence from interfering with the bimolecular fluorescence complementation (BiFC) assay, we prepared protoplasts from etiolated rice seedlings. The coding sequence from *CrNAC036* was inserted into a BiFC expression vector (pCL112) to produce the nYFP vectors. *CrMYB68* was inserted into a BiFC expression vector (pCL113) to produce cYFP vectors (Bhargava *et al.*, 2010). Florescence was observed using a confocal laser-scanning microscope (TCS SP2, Leica, Germany). All the plasmids used in the dual luciferase transcriptional activity assay and the BiFC assay were purified using the QIAGEN Plasmid Midi Kit.

### Yeast two-hybrid analysis

The *CrNAC036* and *CrMYB68* coding sequences were inserted into pGBKT7 and pGADT7 to generate pGBKT7-*CrNAC036* and pGADT7-*CrMYB68* vectors, respectively. A yeast strain (AH109, Clontech) was co-transformed with these two vectors and grown on a selective SD/−Trp/−Leu medium. The interactions were evaluated on SD/−Trp/−Leu/−His/−Ade medium containing X-α-galactosidase.

### Statistical analysis

The variance of the data was analysed using SPSS 16.0 (SPSS Inc. Chicago, IL, USA). Multiple comparisons were performed by one-way ANOVA at the significance level of *P*<0.05 based on Duncan’s multiple range test. Student’s paired *t*-test was performed to assess whether the differences between two genotypes were statistically significant.

### Accession numbers

*CrNAC036* (coding sequence, MH339996), *CrABA4* (promoter sequence, MH339995), and *CrNCED5* (promoter sequence, KY612516) are available at NCBI with the indicated accession numbers. The microarray raw data are available at NCBI’s Gene Expression Omnibus with the accession code of GSE113669.

## Results

### Differences in ABA-associated ripening phenotypes between WT and MT fruit

As described previously, the low ABA content and some late-ripening phenotypes such as the low color index, maturity index, softening, and rotting rate during storage in MT fruit are in accordance with those displayed by other ABA-deficient citrus mutants (Rodrigo *et al.*, 2003; Wu *et al.*, 2014; Zhang *et al.*, 2014; Zhu *et al.*, 2017). To further analyse the effect of ABA on fruit ripening process, we systematically analysed other ABA-associated phenotypes such as the levels of chlorophyll-associated metabolites and photosynthesis-related protein, the subcellular morphology of the chloroplast–chromoplast conversion, and the content of sugars and organic acids.

**Fig. 1.**
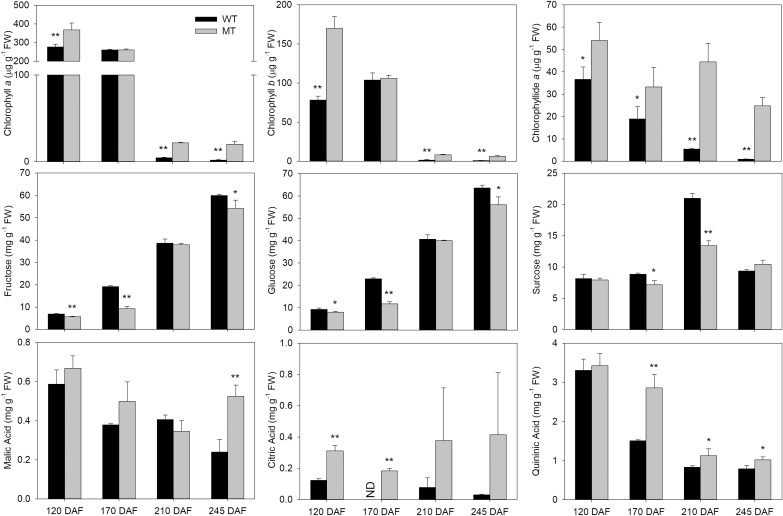
Chlorophyll *a*, chlorophyll *b*, chlorophyllide *a*, sugar, and organic acid levels in the flavedo of WT and MT. The values in each column are the means of three biological replicates. Error bars indicate SD. The asterisks indicate significant differences determined using Student’s *t*-test. *P<0.05; **P<0.01. DAF: days after flowering. ND: not detected.

The WT ripening process was characterized by a decrease in the levels of chlorophyll-related metabolites, such as chlorophyll *a*, chlorophyll *b*, and chlorophyllide *a* (Fig. 1; Supplementary Fig. S1). At the same time, the thylakoid membranes were disassembled and the chloroplasts were gradually converted to chromoplasts, which contained plastoglobules filled with carotenoids. By contrast to WT flavedo, MT flavedo still retained chloroplasts with intact thylakoid membranes at 210 DAF (see Supplementary Fig. S2). Consistently, the MT flavedo contained higher levels of photosystem-associated proteins than the WT flavedo across the ripening process (Supplementary Fig. S3). Moreover, compared with the results of the previous work, similar increases in sugars and decreases in organic acids were also observed in the WT flavedo during fruit ripening in this study; however, the rates of these changes in the MT flavedo were slower than those in the WT flavedo (Fig. 1). Furthermore, we observed the same trend of change in the levels of sugars and organic acids in the flesh of WT and MT fruit (Supplementary Fig. S1).

**Fig. 2.**
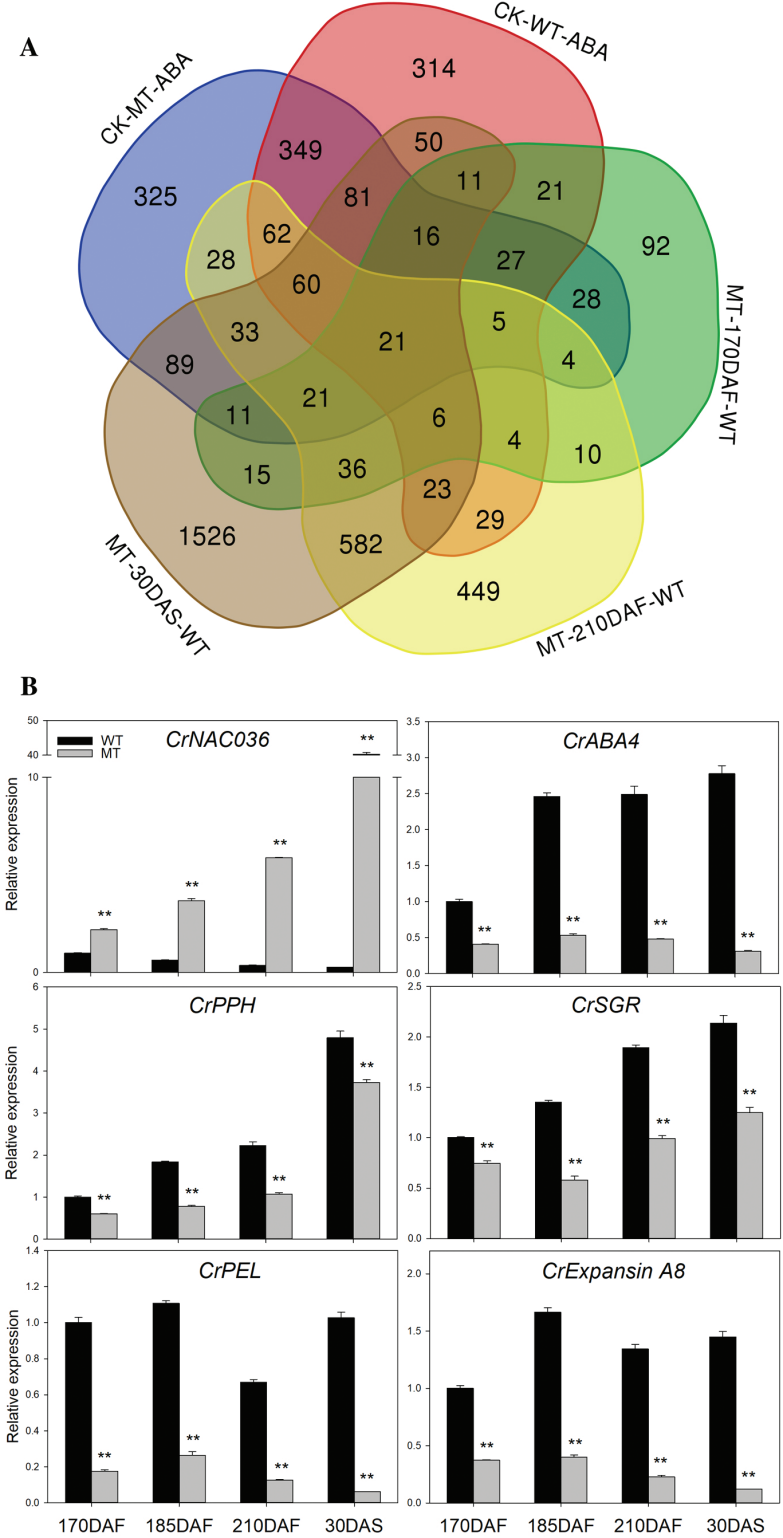
Transcriptome analysis (A) and expression (B) of CrNAC036 and ABA-induced genes at different stages of ripening. (A) CK-MT-ABA and CK-WT-ABA indicate the DEGs of MT and WT between water treatment and ABA treatment, respectively. MT-170DAF-WT, MT-210DAF-WT, and MT-30DAS-WT indicate the DEGs at 170 DAF, 210 DAF, and 30 DAS between MT and WT, respectively. (B) The values in each column are the means of three biological replicates. Error bars indicate SD. The asterisks represent significant differences determined by Student’s *t*-test, **P<0.01. DAF: days after flowering; DAS: days after storage. *CrPEL*, *CrPectate Lyase*; *CrPPH*, *CrPheophytinase*; *CrSGR*, *CrStay-Green*.

To further explore the effect of ABA on ripening-related gene expression and the regulatory mechanism underlying the ABA-deficient phenotype of MT, GeneChip Citrus Genome Arrays (Affymetrix, Santa Clara, CA, USA) were used to characterize change in the transcriptome of MT and WT fruits (170 DAF) after exogenous ABA treatment. Combining the data obtained with former microarray and digital gene expression profiling experiments (Zhu *et al.*, 2017), we analysed the DEGs after ABA treatment and between MT and WT at different fruit stages (Fig. 2A). These analyses indicated that 21 genes were differentially expressed across all transcriptome profiling experiments and the expression of 13 DEGs was consistently higher while one gene was consistently lower in ABA-treated fruits (in comparison with that of water-treated fruit) and WT (in comparison with that of MT at different fruit stage) (Table 1). Among these genes, *CrNCED5* was induced in both WT and MT fruits after ABA treatment and also highly expressed in WT fruit during the fruit development stages. Moreover, some genes involving in ABA-induced ripening process, such as cell wall degradation, were induced by ABA treatment and highly expressed in the WT fruit (Table 1). Interestingly, there were two transcription factors that co-expressed with *CrNCED5* and since NAC family proteins play important roles in both ABA biosynthesis and the fruit ripening process, we inferred that CrNAC036 may be an important regulator of *CrNCED5* (Table 1).

**Table 1.** Consistently differentially expressed genes under ABA treatments and at different ripening stages

Gene	Probe set ID	Transcriptome analysis (log_2_(fold change))					Function	Arabidopsis ortholog
		CK- WT- ABA	CK- MT- ABA	MT- 170DAF- WT	MT- 210DAF- WT	MT- 30DAS- WT		
Ciclev10014639m	Cit.17235.1.S1_s_at	−3.11	−1.07	−1.13	−1.71	−7.81	ABA biosynthesis	AT1G30100 (*AtNCED5*)
Ciclev10029007m	Cit.31377.1.S1_at	1.88	1.98	1.12	3.32	5.50	Transcription factor	AT2G17040 (*AtNAC036*)
Ciclev10029283m	Cit.10057.1.S1_s_at	−1.63	−2.47	−1.52	−1.13	−2.68	Transcription factor	AT2G28500 (*AtLBD11*)
Ciclev10031429m	Cit.35568.1.S1_s_at	−1.25	−1.56	−1.74	−3.39	−4.91	Cell wall degradation	AT1G67750 (*AtPEL*)
Ciclev10032524m	Cit.20839.1.S1_s_at	−1.02	−1.18	−1.11	−1.81	−3.95	Cell wall degradation	AT2G40610 (*AtEXPA8*)
Ciclev10019301m	Cit.2945.1.S1_s_at	−1.59	−2.05	−1.09	−2.42	−4.74	Cell wall degradation	AT1G64390 (*AtGH9C2*)
Ciclev10012384m	Cit.8763.1.S1_s_at	−2.52	−3.60	−1.86	−2.59	−8.10	Water metabolism	AT4G00430 (*AtPIP1;4*)
Ciclev10004103m	Cit.1002.1.S1_s_at	−1.09	−0.96	−1.81	−1.08	−3.00	Glucosinolate metabolism	AT4G31500 (*AtCYP83B1*)
Ciclev10025900m	Cit.15742.1.S1_at	−1.72	−3.34	−1.43	−1.79	−2.16	Lipid metabolism	AT1G75900 (*AtEXL3*)
Ciclev10011714m	Cit.1770.1.S1_at	−1.80	−1.79	−1.88	−3.36	−7.47	Secondary metabolism	AT3G26040
Ciclev10001944m	Cit.22427.1.S1_s_at	−1.93	−2.49	−1.40	−1.19	−3.32	Development, unspecified	AT4G15920
Ciclev10033996m	Cit.26052.1.S1_s_at	−1.06	−1.67	−2.05	−1.72	−4.77	Unknown	AT2G39855
Ciclev10033283m	Cit.21497.1.S1_at	−1.87	−2.26	−1.72	−1.63	−4.12	Unknown	AT2G38905
Ciclev10028078m	Cit.10062.1.S1_at	−1.88	−2.08	−1.14	−1.15	2.91	Amino acid metabolism	AT3G47340(*AtASN1*)
Ciclev10008993m	Cit.1718.1.S1_s_at	3.34	4.15	1.04	1.35	−1.25	Ethylene biosynthesis	AT2G19590 (*AtACO1*)
Ciclev10029695m	Cit.9890.1.S1_s_at	1.80	1.01	−1.17	−1.53	−1.13	Gibberellin-regulated family protein	AT2G14900
Ciclev10005627m	Cit.28626.1.S1_s_at	−1.29	−2.11	−1.83	−1.78	1.72	Cell wall modification	AT1G65680(*ATEXPB2*)
Ciclev10031099m	Cit.4425.1.S1_at	−1.32	2.21	1.54	−1.43	2.25	Cytochrome P450	AT5G05260 (*AtCYP79A2*)
Ciclev10002768m	Cit.12040.1.S1_s_at	−1.36	−2.81	−2.02	−1.81	1.06	Metal handling	AT4G08570
Ciclev10006006m	Cit.165.1.S1_s_at	−1.84	−2.18	−1.23	−3.96	1.76	Light signalling	AT3G22840 (*AtELIP1*)
Ciclev10017113m	Cit.10672.1.S1_s_at	−3.78	−3.28	1.17	−2.02	3.22	Light signalling	AT3G26740 (*AtCCL*)

CK-MT-ABA and CK-WT-ABA indicate the DEGs of MT and WT between water treatment and ABA treatment, respectively. MT-170DAF-WT, MT-210DAF-WT, and MT-30DAS-WT indicate the DEGs at 170 DAF, 210 DAF, and 30 DAS between MT and WT, respectively.

Owing to the slight differences between MT and WT fruit at 120 DAF and in order to acquire more information about the fruit at the breaker and postharvest stages, we analysed the expression of genes involved in ABA biosynthesis and related process (such as chlorophyll and cell wall degradation) using qRT-PCR at 170, 185 and 210 DAF and 30 DAS (Fig. 2B). At 210 DAF, the expression levels of *CrABA4*, *CrPPH* (*CrPheophytinase*), *CrSGR* (*CrStay Green*), *CrPEL* (*CrPectin Lyase*), and *CrExpansin A8* were 4.02-, 1.15-, 1.48-, 27.04-, and 8.74-fold higher in WT fruit than in MT fruit, respectively. By contrast, the expression level of *CrNAC036* in MT fruit was 16.13-fold higher than that in WT fruit.

### Amino acid sequence alignment, subcellular localization, and prokaryotic expression of CrNAC036

**Fig. 3.**
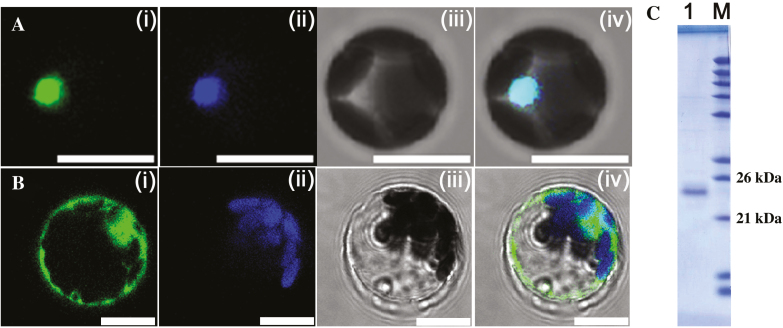
Subcellular localization and prokaryotic expression of CrNAC036. (A) Subcellular localization of CrNAC036. *35S:CrNAC036*-pM999-GFP and *35S:OsGhd7*-CFP were co-transformed into protoplasts. OsGhd7–CFP was used as a nuclear marker. (i) CrNAC036–GFP, (ii) OsGhd7–CFP, (iii) bright field, (iv) merged image. (B) Subcellular localization of pM-999. (i) pM999–GFP, (ii) chlorophyll fluorescence, (iii) bright field, (iv) merged image. The scale bars in (A, B) indicate 10 μm. (C) Prokaryotic expression analysis of His-tagged CrNAC036 with a Coomassie blue-stained 12% SDS gel. Lane 1, His-tagged CrNAC036 protein; M, Marker.

To further analyse the potential function of *CrNAC036*, we compared the amino acid sequence of CrNAC036 with that of 105 Arabidopsis NAC transcription factors. In the phylogenetic analysis, CrNAC036 grouped with the clade containing At2G17040.1, At2G02450.1, At2G02450.2, At5G39820.1, and At2G43000.1 (see Supplementary Fig. S4). Moreover, subcellular localization experiments demonstrated that the CrNAC036–GFP fusion protein was co-localized with a nuclear marker protein (OsGhd7–CFP), indicating that the CrNAC036–GFP fusion protein was accumulated in the nucleus (Fig. 3A).

To determine the DNA binding activity of the CrNAC036 protein, it was expressed in *Escherichia coli* as a 6×His–CrNAC036 fusion protein and purified. We obtained a protein with a molecular mass of ca. 25 kDa (Fig. 3C). MS data indicated that the band corresponded to the N-terminus of the CrNAC036 protein and the a, c and d subdomains that are conserved among NAC transcription factors could all be identified in the fusion protein (see Supplementary Table S4; Supplementary Figs S5, S6). The c and d subdomains are responsible for the specific DNA binding activities of NAC transcription factors (Ooka *et al.*, 2003). Therefore, the purified N-terminus of the CrNAC036 protein could be utilized for analysing the DNA binding activity of CrNAC036.

### CrNAC036 specifically repressed the expression of *CrNCED5*

**Fig. 4.**
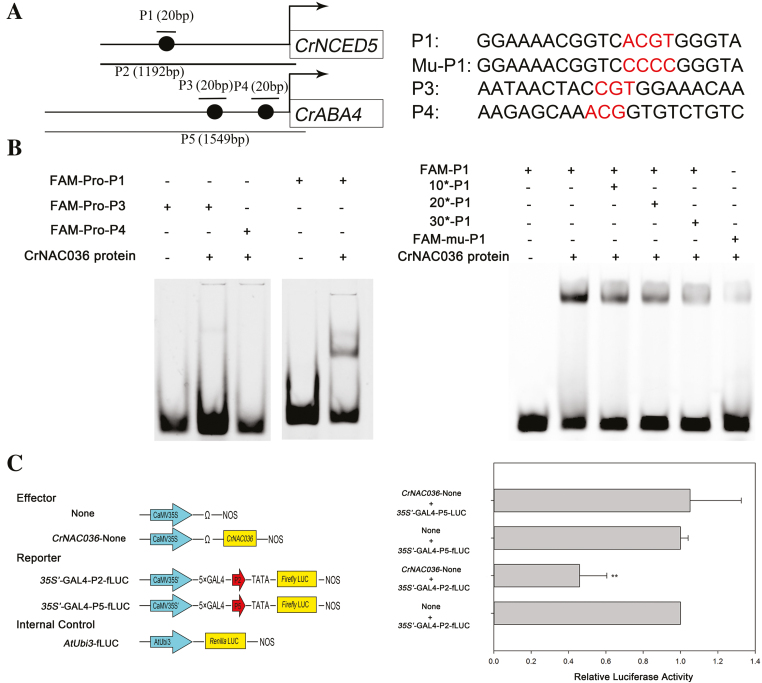
EMSA and dual luciferase assay. (A) Schematic diagram of the promoter model and sequences. The sequences used in the EMSA and dual luciferase assay are indicated on the left; the sequences used in the EMSA are indicated on the right. Black circle indicates the core-binding motif of NAC family. (B) Binding of CrNAC036 to the promoters of *CrNCED5* and *CrABA4*. In EMSA, 10-, 20-, and 30-fold excess of non-labeled probes were used as competitors. (C) Diagram of the constructs used in the dual luciferase assays and the regulation of CrNAC036 on *CrNCED5* and *CrABA4*. The fLUC/rLUC ratio represents the relative activity of the *CrNCED5* and *CrABA4* promoters. The values in each column are the means of three biological replicates. Error bars indicate the SD. The double asterisks represent statistically significant differences determined using Student’s *t*-test (**P<0.01).

Previous studies reported that the senescence-associated NAC transcription factor family can bind to the DNA sequences containing the conserved sequence motif CGT/ACG (Podzimska-Sroka *et al.*, 2015). We found the CGT/ACG motif in the promoters of *CrNCED5* and *CrABA4* (Fig. 4A). To test whether CrNAC036 can bind to the promoters of *CrNCED5* and *CrABA4*, the purified 6×His–CrNAC036 fusion protein was incubated with 20-bp probes containing the CGT/ACG sequence from the promoter regions of *CrNCED5* and *CrABA4*. It was found that the CrNAC036 protein did not bind to the two probes containing the CGT/ACG motifs from the *CrABA4* promoter in the EMSA. However, the P1 probe from the *CrNCED5* promoter was specifically bound by the CrNAC036 protein, as indicated by the retardation of its mobility in the EMSA. Furthermore, although the CrNAC036 protein bound the mutant probe derived from P1, the binding activity was considerably lower than that observed for the wild-type P1 probe (Fig. 4B).

To further evaluate whether CrNAC036 affected the activities of the *CrNCED5* and *CrABA4* promoters, we fused the *CrNCED5* and *CrABA4* promoters including the core-binding motif of NAC family (i.e. the CGT/ACG sequence) to the firefly luciferase reporter gene, and these were then transiently co-expressed in protoplasts. Results indicated that CrNAC036 significantly repressed the activity of the *CrNCED5* promoter but did not affect that of the *CrABA4* promoter (Fig. 4C). Based on the results of EMSA and dual-luciferase experiments, it can thus be inferred that CrNAC036 can specifically bind to the promoter of *CrNCED5* and represses its activity.

### CrNAC036 interacted with CrMYB68 to synergistically down-regulate the expression of *CrNCED5*

**Fig. 5.**
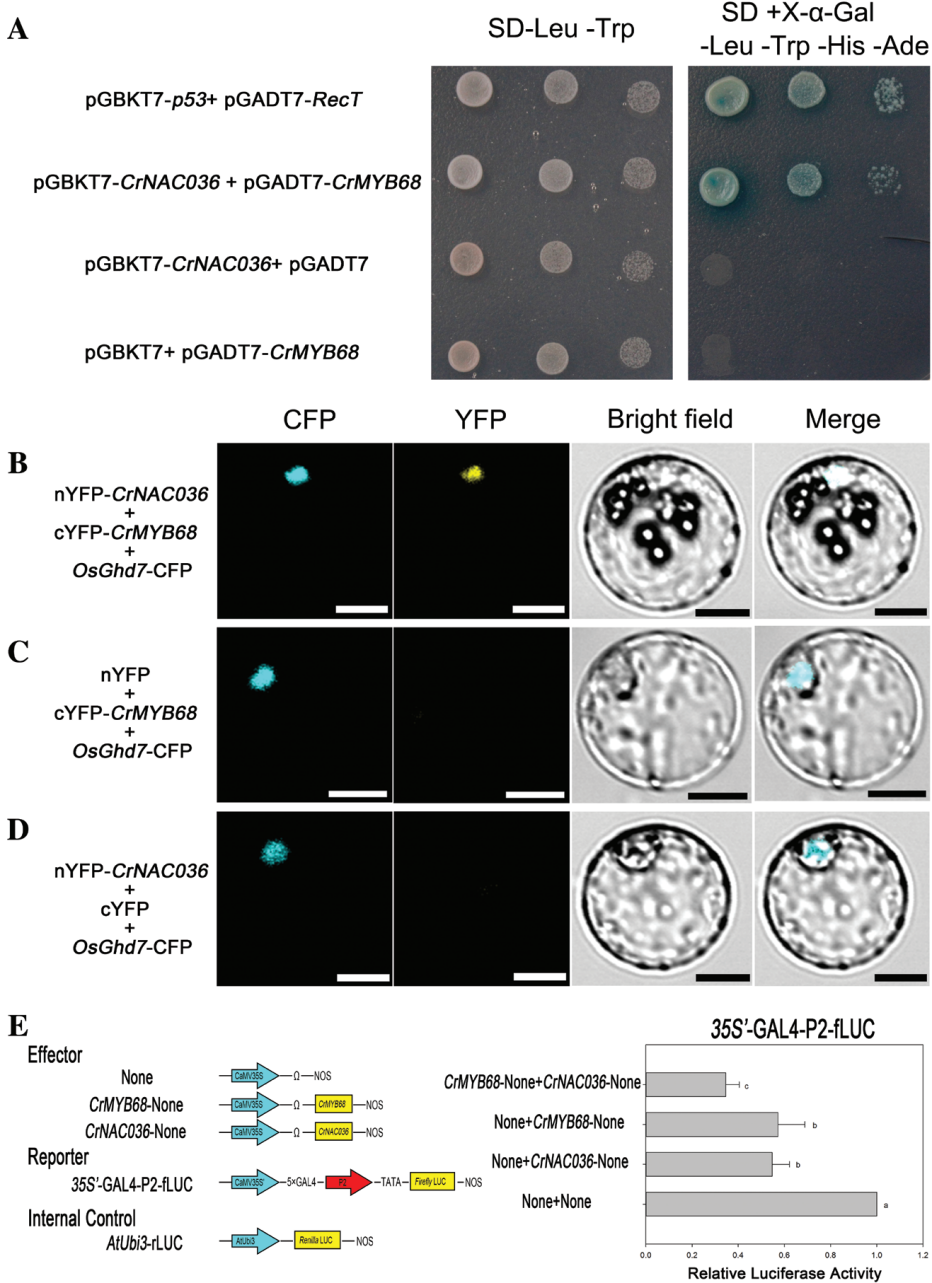
Interactions between CrNAC036 and CrMYB68 and their cooperative regulation of the *CrNCED*5 promoter. (A) Interactions between CrNAC036 and CrMYB68 in the yeast two-hybrid assays. Blue colonies growing on a synthetic drop-out medium lacking Trp, Leu, His, and Ade (SD/−Trp/−Leu/−His/−Ade) and containing X-α-galactosidase indicate protein–protein interactions. Co-transformation of pGBKT7-*p53* and pGADT7-*RecT* was used as a positive control. (B–D) Nuclear interactions of CrNAC036 with CrMYB68. Confocal images of transiently transformed nYFP–CrNAC036 and cYFP–CrMYB68 generating YFP signal in nucleus (OsGhd7–CFP was used as a nuclear marker) (B). Interaction with empty vectors was not observed (C, D). Scale bars: 10 μm. (E) Dual luciferase assays in protoplasts co-expressing *CrNAC036* and *CrMYB68*. The fLUC/rLUC ratio represents the relative activity of the *CrNCED5* promoter (P2). The sequence of P2 is indicated in Fig. 4A. The values in each column are the means of three biological replicates. Error bars indicate SD. Different letters indicate significant differences according to Duncan’s test (*P*<0.05).

NAC proteins usually enhance their own transcriptional activities via interaction with other transcription factors (Olsen *et al.*, 2005). Our previous study indicated that *CrMYB68* displays a similar expression pattern to *CrNAC036* in the MT fruit and can also directly regulate *CrNCED5* (Zhu *et al.*, 2017). In order to determine whether CrNAC036 can interact with CrMYB68 to synergistically regulate the expression of *CrNCED5*, we first tested whether CrMYB68 and CrNAC036 can interact in the yeast two-hybrid system and BiFC assays. The yeast strains harboring both the pGADT7-*CrMYB68* and the pGBKT7-*CrNAC036* vectors could grow and exhibit blue color on a medium containing X-α-Gal without leucine, tryptophan, histidine, and adenine. These results indicated that CrNAC036 can interact with CrMYB68 in the yeast two-hybrid system (Fig. 5A). The ability of these proteins to interact with each other was independently verified by BiFC experiments. As shown in Fig. 5B, the interaction between CrNAC036 with the C-terminus and CrMYB68 with the N-terminus of yellow fluorescent protein (YFP) yielded a fluorescence signal in the nucleus (Fig. 5B).

Secondly, we performed a dual-luciferase experiment by transiently co-expressing the two effector vectors and a luciferase reporter gene in protoplasts. As a result, the combinations of ‘None (empty vector)+*CrNAC036*-None’ and ‘None (empty vector)+*CrMYB68*-None’ significantly repressed the activity of the promoter from *CrNCED5*. However, the activity of the *CrNCED5* promoter was the most obviously reduced when ‘*CrNAC036*-None’ and ‘*CrMYB68*-None’ were co-expressed (Fig. 5E). These results indicated that the protein–protein interactions between CrNAC036 and CrMYB68 do synergistically affect the expression of *CrNCED5*.

## Discussion

ABA is one of the key regulators of the fruit ripening process. The transcriptional regulation of ABA biosynthetic genes has been well studied in fruits (Endo *et al.*, 2016; Luo *et al.*, 2017; Wang *et al.*, 2019b). However, comprehensive details of the effect of ABA on citrus ripening and the regulation of ABA biosynthesis in citrus by a multi-transcription factor complex remain largely unknown. Here, we performed metabolic, cytological, and transcriptome analysis of an ABA-deficient mutant from *Citrus reticulata* cv. *Suavissima*. It was found that ABA served as an important regulator of citrus ripening and its biosynthesis was under the synergistic regulation of CrNAC036 and CrMYB68 by suppressing the expression of *CrNCED5*.

### ABA plays an important role in the ripening process of citrus fruit

In MT fruit, some ripening parameters (such as color index, maturity index, and softening) were significantly delayed, which was in accordance with those displayed by other ABA-deficient citrus mutants (Rodrigo *et al.*, 2003; Wu *et al.*, 2014; Zhang *et al.*, 2014; Zhu *et al.*, 2017). Moreover, in this study, the delay of chlorophyll degradation, the chloroplast–chromoplast transition, and sugar and organic acid metabolism in MT further indicated that ABA can systematically affect the citrus fruit ripening process (Fig. 1; Supplementary Fig. S1). Furthermore, many studies have reported that ABA can directly promote ripening-related process, such as chlorophyll degradation, carotenoid biosynthesis and softening, by regulating gene expression (Zaharah *et al.*, 2013; Gao *et al.*, 2016; Rehman *et al.*, 2018). These effects of ABA could also be confirmed in MT fruit, with the expression of genes that contribute to chlorophyll degradation and cell wall modification (i.e. *CrPEL*, *CrExpansin A8*, *CrPPH*, and *CrSGR*) being consistently lower in MT fruit than in WT fruit during the ripening process and the demonstration that the expression of *CrPEL* and *CrExpansin A8* could be significantly induced by exogenous ABA treatment (Fig. 2). Additionally, injection of ABA caused an increase in the glucose and fructose levels of citrus fruit and exogenous ABA treatment can accelerate fruit coloring of citrus (Kojima *et al.*, 1995; Wang *et al.*, 2016; Rehman *et al.*, 2018). Taken together, all of the previously published work and our own findings indicate that ABA is an essential positive regulator of ripening of citrus fruits.

### CrNAC036 plays an important role in ABA-deficient phenotype of MT

Our results from the transcriptome and qRT-PCR experiments indicate that the expression of *CrNCED5* and *CrABA4* was robustly lower in MT than that in WT (Fig. 2; Table 1; Zhu *et al.*, 2017). We also checked the expression of other genes involved in ABA biosynthesis but found that their expression did not vary robustly (Zhu *et al.*, 2017). Thus, as NCED5 was the dominant NCED for the ABA biosynthesis in *C. clementina* flavedo compared with other NCEDs (Agustí *et al.*, 2007), the low expression of *CrNCED5* and *CrABA4* may lead to the low ABA level in MT. Moreover, we observed, in the transcriptome data, that the expression of *CrNAC036* displayed a significantly negative correlation to that of *CrNCED5* and *CrABA4* (Table 1; Fig. 2). Additionally, we found that the amino acid sequence of CrNAC036 is similar to that of JUB1, which inhibits Arabidopsis senescence (see Supplementary Fig. S4), and note that the ABA content of *jub1-1* plants was significantly increased compared with that of the wild type (Wu *et al.*, 2012). Furthermore, DAP-seq analysis indicated that JUB1 can directly bind to the promoter of *AtNCED5* (O’Malley *et al.*, 2016). When taken together these data suggest that CrNAC036 may negatively regulate ABA biosynthesis by down-regulating the expression of *CrNCED5* or *CrABA4*. However, whilst conserved NAC-recognition sequences were recognized in the promoters of both *CrNCED5* and *CrABA4*, the data from the EMSA indicated that CrNAC036 can specifically bind to the promoter of *CrNCED5* but not to that of *CrABA4* (Fig. 4). Moreover, the data from the dual luciferase assay indicated that CrNAC036 can repress the activity of the *CrNCED5* promoter while not affecting the activity of the *CrABA4* promoter (Fig. 4). Based on these results, it can be concluded that CrNAC036 is an important regulator of ABA biosynthesis in citrus fruit, a task it achieves by specific down-regulation of the expression of *CrNCED5*.

Transcription factors and hormones such as ABA and ethylene substantially influence fruit ripening. Several transcription factor families have been proven to promote ripening through affecting ABA (ABF2 in grape) or ethylene (RIN, CNR in tomato and MdMYC2 in *Malus domestica*) metabolism and signaling (Klee and Giovannoni, 2011; Nicolas *et al.*, 2014; Li *et al.*, 2017b). However, until recently no transcription factor family had been reported to regulate both fruit ABA and ethylene metabolism. Recently, Lü *et al.* (2018) reported that carpel senescence-related NAC transcription factors play vital roles in the ripening process of both climacteric fruit without recent whole-genome duplication and non-climacteric fruit. In tomato, SlNAC19/48 can directly induce the expression of *ACO1* and *ACS2* and in *Musa acuminata*, MaNAC1/2 can affect ethylene signaling by interacting with EIN3 (Shan *et al.*, 2012; Ma *et al.*, 2014a; Kou *et al.*, 2016). In the present study, our findings concerning the transcriptional regulation of CrNAC036 greatly improves our understanding of the transcriptional regulation of ABA metabolism in fruit. They additionally enrich our knowledge concerning the importance of regulators of the NAC transcription factor family on the biosynthesis of two hormones known to be vital for fruit ripening.

### CrNAC036 and CrMYB68 synergistically inhibit ABA biosynthesis by down-regulating the expression of *CrNCED5*

Owing to their sessile nature, plants have evolved complex mechanisms to activate or repress gene expression in order to adapt to their prevailing environments. As important regulators of gene expression, besides working alone, different transcription factors can form complexes and recognize *cis*-regulatory elements within a target gene promoter for synergistic or antagonistic regulation of gene transcription. Given that ABA is an important regulator of stress tolerance and the fruit ripening process, the transcriptional regulation of *NCEDs* by a single transcription factor has been reported in many crops and fruits (Jiang *et al.*, 2012; Finkelstein, 2013; Jensen *et al.*, 2013; Endo *et al.*, 2016; Zong *et al.*, 2016; Luo *et al.*, 2017; Ma *et al.*, 2018; Wang *et al.*, 2019b). However, to date research concerning synergistic regulation of fruit *NCEDs* by transcription factor complexes remains rare.

In previous publications, transcription factors of the NAC family have been reported to interact with distinct types of transcription factors including NAC family proteins, WRKY family proteins, TCP family proteins, and zinc finger-containing transcription factors (Olsen *et al.*, 2004; Tran *et al.*, 2007; Wang *et al.*, 2015; Zhou *et al.*, 2015; Li *et al.*, 2017a). Moreover, our earlier research demonstrated that *CrMYB68* was highly expressed in the MT fruit and the protein can directly repress the expression of *CrNCED5* (Zhu *et al.*, 2017). In the present study, *CrNAC036* was revealed to be highly co-expressed with *CrMYB68*, and Y2H and BiFC results indicated that CrNAC036 physically interacted with CrMYB68. Given that both CrNAC036 and CrMYB68 are repressors of *CrNCED5*, the dual luciferase assay co-expressing these two transcription factors showed lower promoter activity of *CrNCED5* compared with that of the single-expression dual luciferase assay, which indicated that CrNAC036 interacts with CrMYB68 in order to synergistically represses the expression of *CrNCED5* (Fig. 5).

When taken together, the results of our study thus highlight the powerful effect of ABA on the citrus fruit ripening process as well as providing considerable insight into the regulation of *CrNCED5* by CrNAC036 either singularly or synergistically with CrMYB68. These results broaden our understanding of the function of NAC family transcription factors with regard to the key regulators (ABA and ethylene) of non-climacteric and climacteric fruit ripening processes and of the intricate regulatory network of transcription factor complexes in ABA biosynthesis.

## Supplementary data

Supplementary data are available at *JXB* online.

Fig. S1. Pheophorbide *a* and pheophytin *a* levels in the flavedo and sugar and organic acid levels in the flesh of WT and MT.

Fig. S2. Transmission electron microscopy analysis of the morphological changes in the flavedo plastids from WT and MT.

Fig. S3. Levels of photosystem-associated proteins in the flavedo at four developmental stages.

Fig. S4. Phylogenetic analysis of CrNAC036 and NAC proteins from Arabidopsis.

Fig. S5. Peptides from CrNAC036 identified using MALDI-TOF/TOF MS.

Fig. S6. The MS/MS spectrum of the identified peptides of CrNAC036 protein.

Table S1. The DEGs of MT and WT after ABA treatment

Table S2. Primers used in this study.

Table S3. Names and TAIR ID numbers of 105 NAC transcription factors from Arabidopsis.

Table S4. Peptides identified by 6×His–CrNAC036 protein using the MALDI-TOF/TOF MS method.

eraa118_suppl_Supplementary_file001

eraa118_suppl_Supplementary_file002
