# Supplementary material for: A NAC transcription factor and its interaction protein hinder abscisic acid biosynthesis by synergistically repressing NCED5 in Citrus reticulata
Source: J Exp Bot. 2020 May 31;71(12):3613–25. doi: 10.1093/jxb/eraa118 (PMC7475259; doi:10.1093/jxb/eraa118)
Supplement: eraa118_suppl_Supplementary_file001 [file eraa118_suppl_supplementary_file001.pdf]

Fig. S1

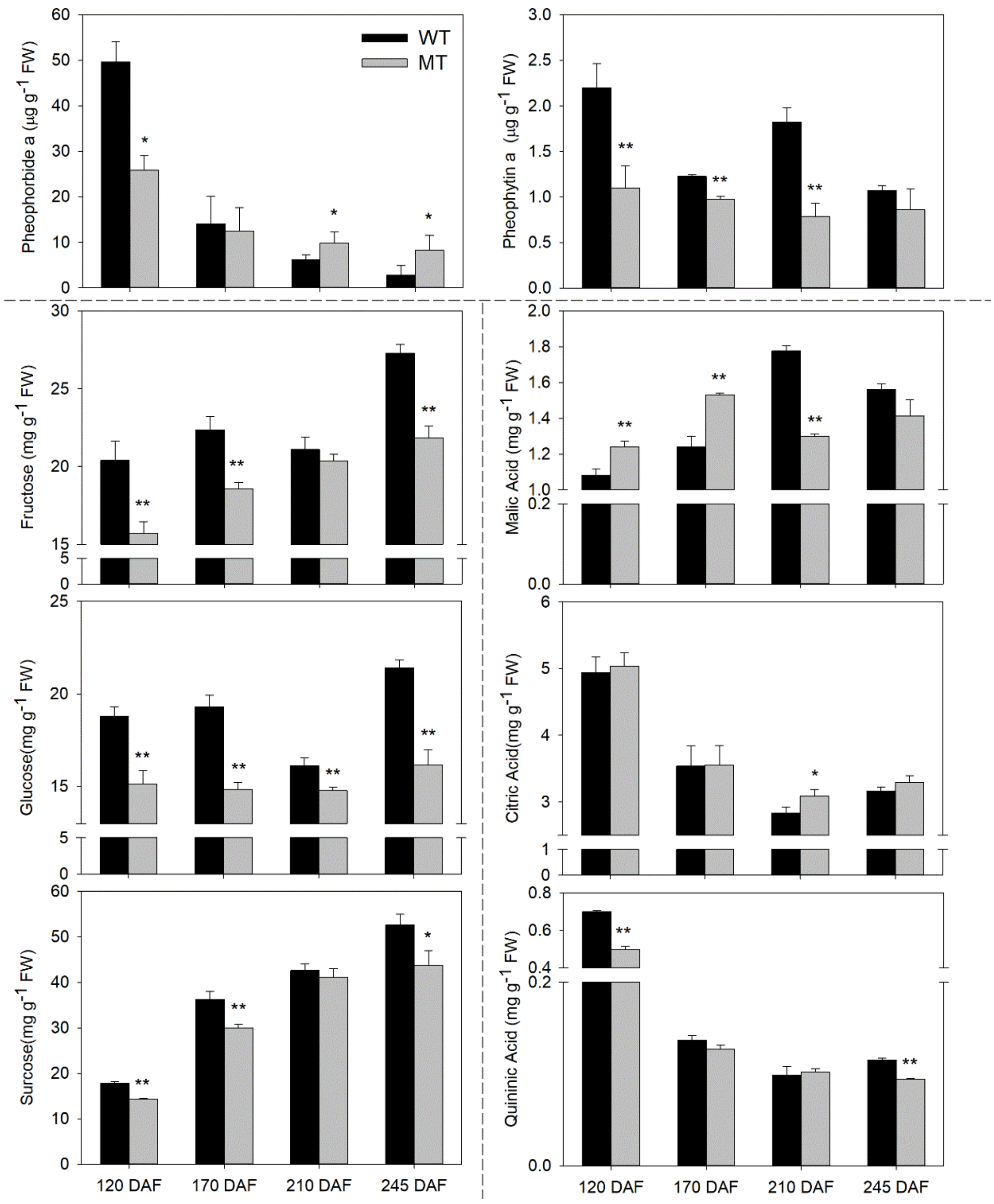
WT
MT
Pheophorbide a (μg g-1 FW)
Pheophytin a (μg g-1 FW)
Fructose (mg g-1 FW)
Malic Acid (mg g-1 FW)
Glucose(mg g-1 FW)
Citric Acid(mg g-1 FW)
Surcose(mg g-1 FW)
Quininic Acid (mg g-1 FW)
120 DAF
170 DAF
210 DAF
245 DAF

Fig. S2

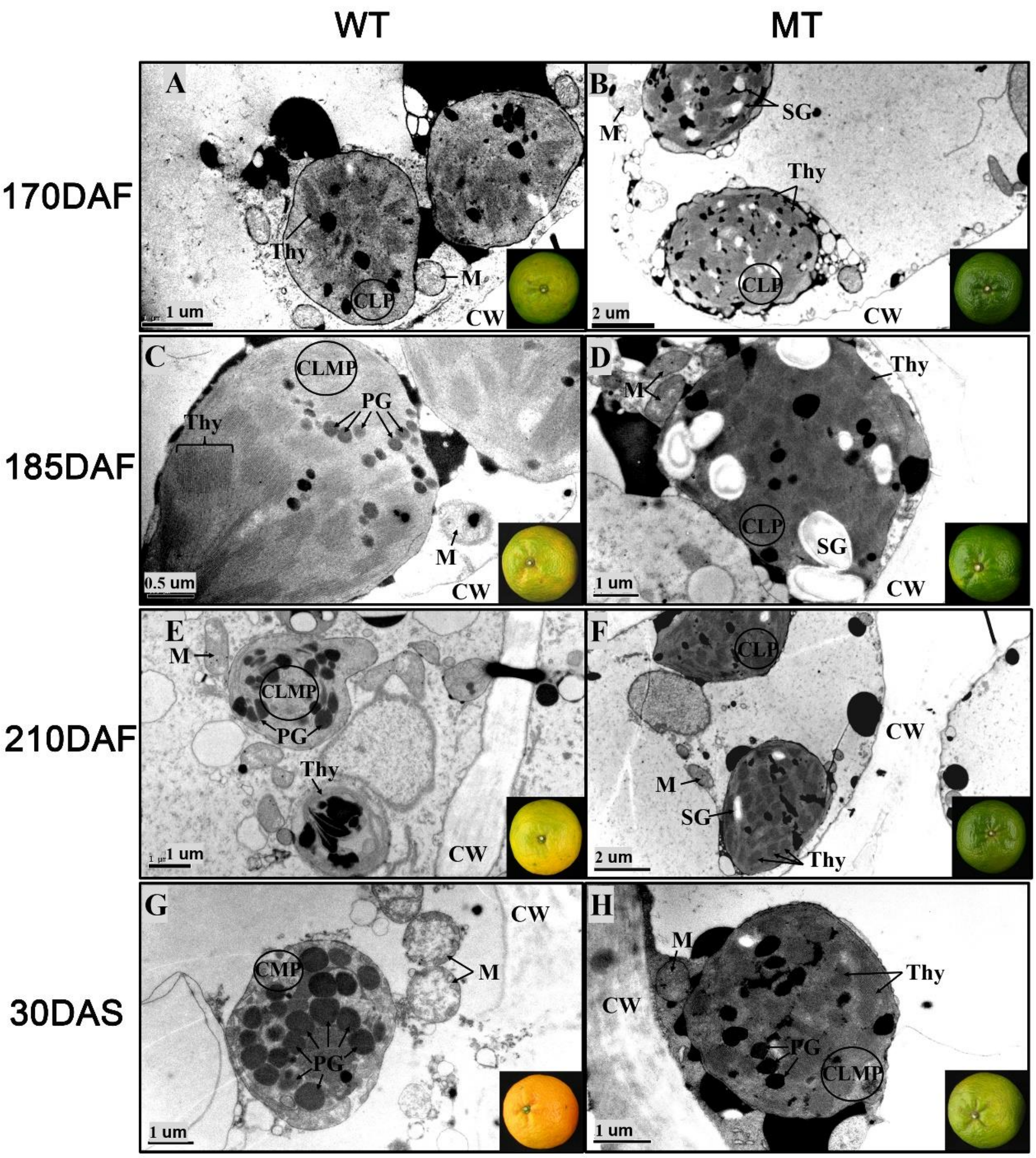
WT
MT
170DAF
185DAF
210DAF
30DAS
A
Thy
CLP
M
CW
1 um
B
M
SG
Thy
CLP
CW
2 um
C
CLMP
PG
Thy
M
CW
0.5 um
D
M
Thy
CLP
SG
CW
1 um
E
M
CLMP
PG
Thy
CW
1 um
F
CLP
CW
M
SG
Thy
2 um
G
CW
CMP
M
PG
1 um
H
M
Thy
CW
PG
CLMP
1 um

Fig. S3

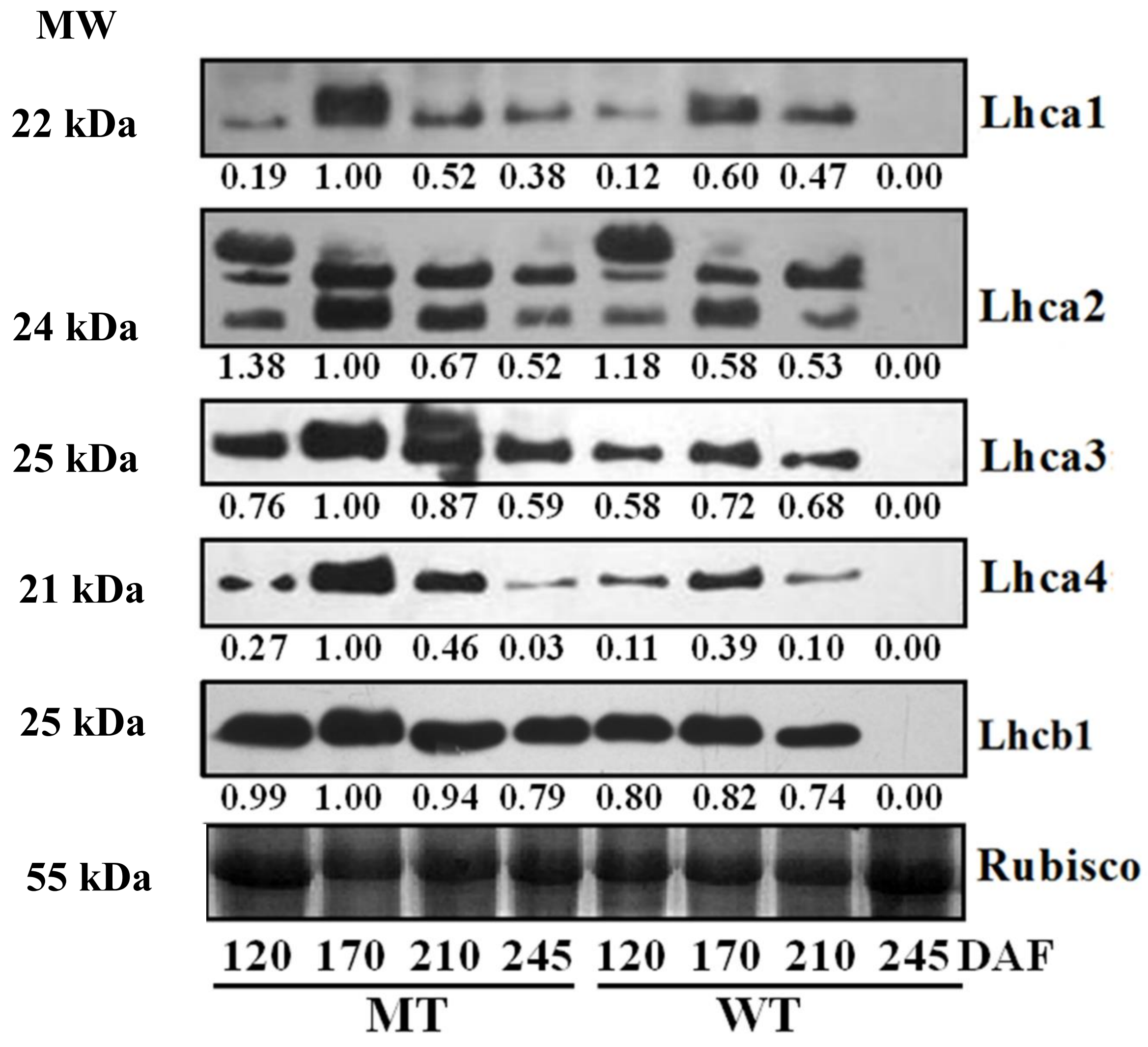
MW
22 kDa
24 kDa
25 kDa
21 kDa
25 kDa
55 kDa
Lhca1
0.19 1.00 0.52 0.38 0.12 0.60 0.47 0.00
Lhca2
1.38 1.00 0.67 0.52 1.18 0.58 0.53 0.00
Lhca3
0.76 1.00 0.87 0.59 0.58 0.72 0.68 0.00
Lhca4
0.27 1.00 0.46 0.03 0.11 0.39 0.10 0.00
Lhcb1
0.99 1.00 0.94 0.79 0.80 0.82 0.74 0.00
Rubisco
120 170 210 245 120 170 210 245 DAF
MT
WT

Fig. S4

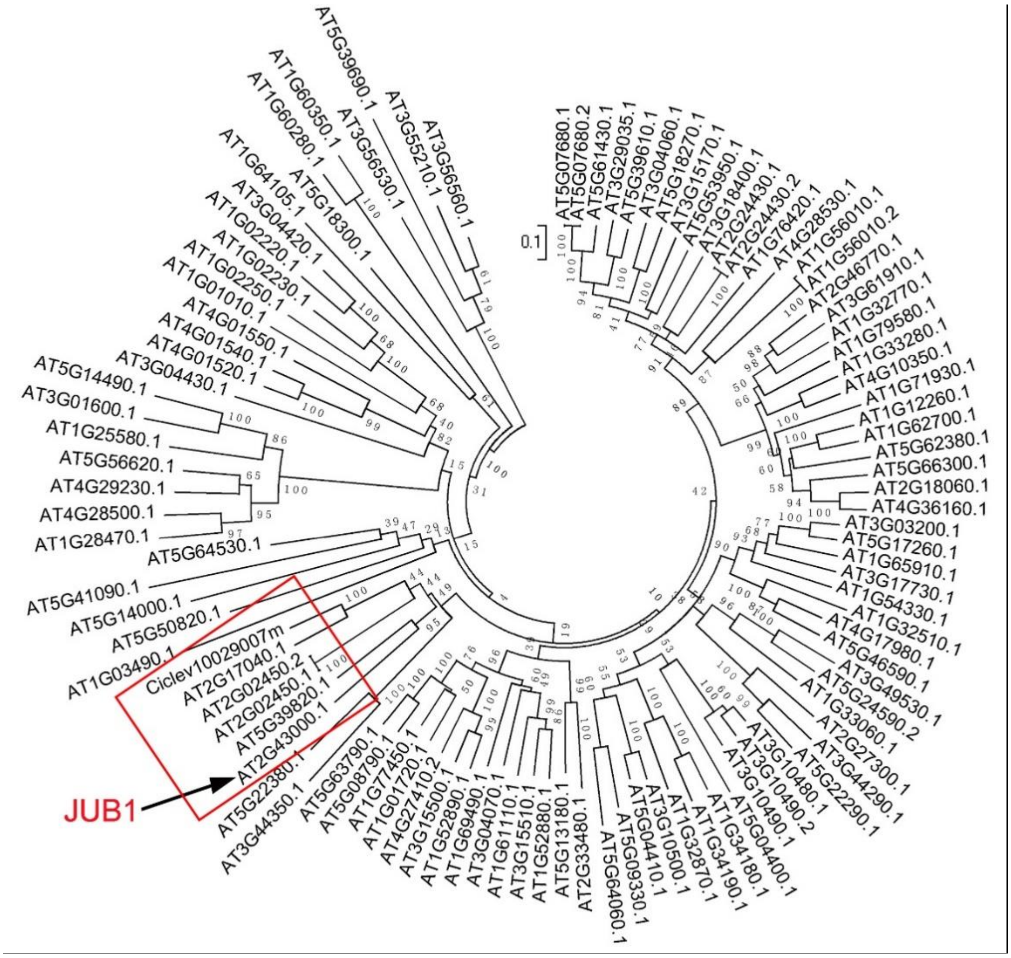
0.1
AT5G07680.1
AT5G07680.2
AT5G61430.1
AT3G29035.1
AT5G39610.1
AT3G04060.1
AT5G18270.1
AT3G15170.1
AT5G53950.1
AT3G18400.1
AT2G24430.1
AT2G24430.2
AT1G76420.1
AT4G28530.1
AT1G56010.1
AT1G56010.2
AT2G46770.1
AT3G61910.1
AT1G32770.1
AT1G79580.1
AT1G33280.1
AT4G10350.1
AT1G71930.1
AT1G12260.1
AT1G62700.1
AT5G62380.1
AT5G66300.1
AT2G18060.1
AT4G36160.1
AT3G03200.1
AT5G17260.1
AT1G65910.1
AT3G17730.1
AT1G54330.1
AT1G32510.1
AT4G17980.1
AT5G46590.1
AT3G49530.1
AT5G24590.2
AT1G33060.1
AT2G27300.1
AT3G44290.1
AT5G22290.1
AT3G10480.1
AT3G10490.1
AT3G10490.2
AT5G04400.1
AT1G34180.1
AT1G34190.1
AT1G32870.1
AT3G10500.1
AT5G04410.1
AT5G09330.1
AT5G64060.1
AT2G33480.1
AT5G13180.1
AT1G52880.1
AT3G15510.1
AT1G61110.1
AT3G04070.1
AT1G69490.1
AT1G52890.1
AT3G15500.1
AT4G27410.2
AT1G01720.1
AT1G77450.1
AT5G08790.1
AT5G63790.1
AT3G44350.1
AT5G22380.1
AT2G43000.1
AT5G39820.1
AT2G02450.1
AT2G02450.2
AT2G17040.1
Ciclev10029007m
AT1G03490.1
AT5G50820.1
AT5G14000.1
AT5G41090.1
AT5G64530.1
AT1G28470.1
AT4G28500.1
AT4G29230.1
AT5G56620.1
AT1G25580.1
AT3G01600.1
AT5G14490.1
AT3G04430.1
AT4G01520.1
AT4G01540.1
AT4G01550.1
AT1G01010.1
AT1G02250.1
AT1G02230.1
AT1G02220.1
AT3G04420.1
AT1G64105.1
AT1G60280.1
AT1G60350.1
AT5G18300.1
AT3G56530.1
AT5G39690.1
AT3G55210.1
AT3G56560.1
JUB1

Fig. S5

a-subdomain **b-subdomain** **c-subdomain**

MTPELELPGFR**FHPTEDELLNFYLR**NIVFGKKSHFDIIGFLNIYHHDPWELPGLSKIGER**EWYFFVPR**DRKHGS

c-subdomain **d -subdomain**

GGRPNRTTEHGFWKATGSDRKIVSLSDPKRVIGLRK**TLVFYEGR**APKGHK**TDWVMNEYR**LPDGCPLP

e-subdomain

KDIVLCKIYRKATSLKVLEQRAAMEEDQMKTSFTSPSPPSPTLDTSSFCGQPEELLGPISLPHVMLKQEEQEVSMVQGTYTEEKTVSTAT

SLQLPQGKINLPELQVPKMNTDWSQDPIWSQLSPWLQNIAQNLTPYASILN-

Fig. S6

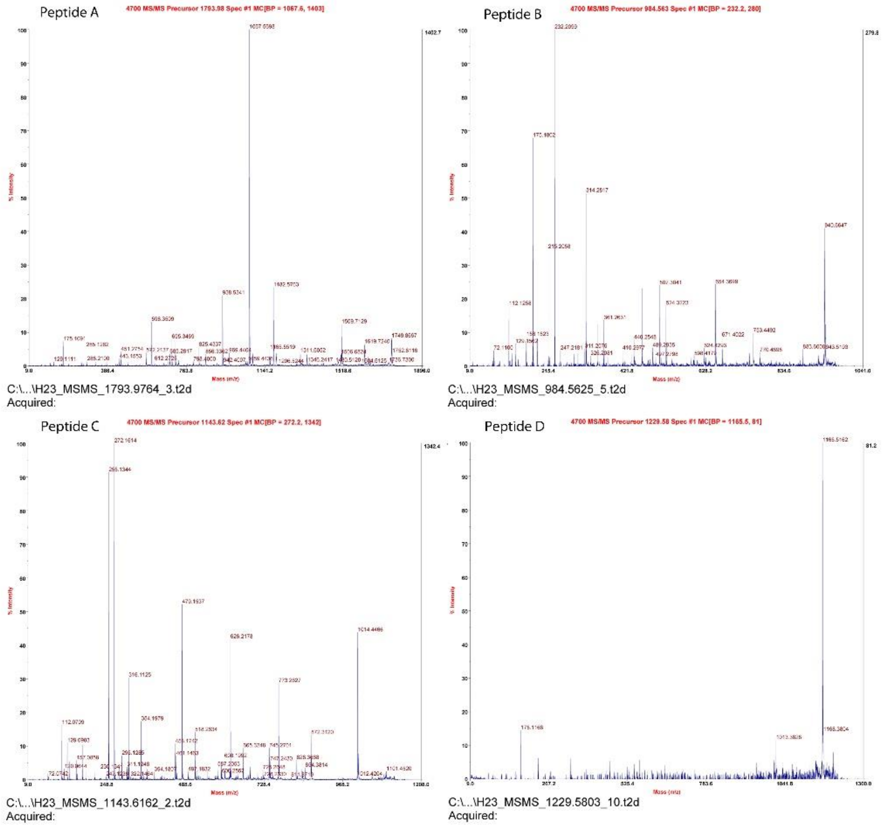

Peptide A
C:\...\H23_MSMS_1793.9764_3.t2d
Acquired:
Peptide B
C:\...\H23_MSMS_984.5625_5.t2d
Acquired:
Peptide C
C:\...\H23_MSMS_1143.6162_2.t2d
Acquired:
Peptide D
C:\...\H23_MSMS_1229.5803_10.t2d
Acquired:
